# Results of Type I Tympanoplasty Using Fascia with or without Cartilage Reinforcement: 10 Years’ Experience

**Published:** 2018-03

**Authors:** Ali Kouhi, Mohammad Taghi Khorsandi Ashthiani, Mir Mohammad Jalali

**Affiliations:** 1 ***Otorhinolaryngology Research Center, Tehran University of Medical Sciences, Tehran, Iran.***; 2 *Rhino-Sinus, Ear, and Skull Base Diseases Research Center,* *Guilan University of Medical Sciences, Rasht, Iran.*

**Keywords:** Ear cartilage, Fascia, Hearing, Otitis media, Tympanoplasty, Treatment outcome

## Abstract

**Introduction:**

There remains controversy about the optimal kind of graft to repair tympanic membrane. The purpose of this study was to evaluate the anatomical and auditory outcomes of type I tympanoplasty using fascia with or without cartilage reinforcement.

**Materials and Methods:**

This retrospective cohort study was conducted from 2005 to 2015. All cases were surgically treated by a single surgeon. We excluded cases in which the etiology of chronic otitis media was cholesteatoma. According to the use of cartilage reinforcement in the posterosuperior part of the graft, patients were divided into two groups, and the results of anatomical and auditory evaluation were compared between the two groups. The anatomical outcome was grafting success and the auditory outcome was improvement of air bone gap (ABG).

**Results:**

A total of 320 patients were classified in Group A (tympanoplasty with fascia temporalis only) and 346 were in Group B (tympanoplasty with cartilage reinforcement). All patients were followed for at least 2 years. The overall success rate in the two groups was 91.6% and 93.4%, respectively (P=0.3). The most common cause of failure in the two groups was re-perforation (5.6% and 3.8%, respectively). The improvement of ABG in two groups was 18.5 dB and 3.2 dB, respectively. The difference between two groups was statistically significant (P<0.001).

**Conclusion:**

In patients with dry perforation of the tympanic membrane, the anatomical success with tympanoplasty with fascia only or with cartilage reinforcement was similar. However, hearing improvement in the fascia only group was greater than in the group undergoing cartilage reinforcement.

## Introduction

 The use of a temporalis fascia graft is standard for the reconstruction of tympanic membrane (1,2). However, fascia temporalis contains irregular elastic fibers and fiber connective tissue. Hence, the results of tympanoplasty cannot be predicted (3). On the other hand, cartilage is resistant to inflammation and infection and sustains its shape for a long time. Also cartilage has a stable shape and is tighter than the fascia and does not contain fibrous tissue, so the result after surgery is more predictable (4). A cartilage graft can be used as a perichondrium cartilage island, palisade or together with fascia temporalis (5). The graft can be strengthened by placing the cartilage graft in the upper posterior part and medial to the fascia temporalis. Although cartilage prevents recurrence of the retraction pocket, there is concern about the impairment of the tympanic membrane vibration.

The literature contains conflicting findings in this regard (3,6–9). Kalcioglu et al. (7) showed that no statistically significant difference between cartilage and fascia in the short-term and long-term follow-up. In a retrospective study, Uslu et al. described the results of cartilage reinforcement in 60 patients (8). In 47 cases, tympanic membrane closure occurred. After surgery, the hearing thresholds improved significantly. Tek et al (3) compared the result of tympanoplasty with the anterior cartilage reinforcement technique (37 cases) and tympanoplasty with fascia only (40 cases). There was a statistically significant difference in the rate of graft ingrowth in the two groups. Closure of tympanic membrane was observed in 100% and 66% cases in the groups, respectively. However, there was no significant difference in the auditory results between the two groups. Khan et al. (9) published the results of 28 patients with large perforation (>50%) tympanic membrane. In this prospective study, fascia temporalis was enhanced with sliced ​​tragal cartilage, and the rate of closure was 100%. The mean air bone gap (ABG) after the operation was 9.64 dB. Despite these findings, some surgeons believe that retraction and perforation can still occur in other parts of the tympanic bone, not reinforced with cartilage (10). Articles about the results of cartilage reinforcement with type I tympanoplasty are scarce. The purpose of this study was to compare the anatomical and auditory effects of this method with fascia temporal graft only.

## Materials and Methods

This retrospective cohort study was conducted from 2005 to 2015. The records of patients undergoing ear surgery in a university hospital and a private center were reviewed. According to the use of cartilage reinforcement in the posterior upper limb, patients were divided into two groups: A) patients undergoing type 1 tympanoplasty using fascia temporalis only; B) patients undergoing tympanoplasty using cartilage reinforcement. The results of an anatomical and auditory evaluation were compared between the two groups. Anatomical outcome was success in grafting and auditory outcome was improvement of ABG. The ABG was calculated by averaging the difference in air conduction bone conduction hearing thresholds at frequencies of 0.5, 1, 2, and 4 kHz.

Inclusion criteria were chronic otitis media (COM) patients undergoing tympanoplasty type I, having dry perforation for at least 1 month before surgery, aged over 16 years, minimum follow-up of 6 months, and primary surgery. Chronic otitis media was defined as an inflammatory process in the middle-ear space that results in long-term and permanent changes in the tympanic membrane. We excluded cases in which the etiology of COM was cholesteatoma.

All surgeries were performed by a surgeon (KHM) under general anesthesia. The technique used in all patients was underlay grafting. In Group B, the cartilage graft was placed on the medial side of the upper posterior portion of the fascia temporalis. Cephalexin was given during the postoperative period.

The ear dressing was removed 3 days after surgery. The first postoperative microscopic examination of the ear was performed 1 month after surgery. The presence of an intact graft with an aerated middle ear cleft at the end of 6 months was considered a success. To assess the effect of the cartilage reinforcement technique on auditory function, the hearing threshold gain was assessed only in patients whose graft was anatomically intact. To analyze continuous variables between the two groups and within a group, a t-test and paired t-test were used, respectively. The chi-square test was used to compare categorical variables in the two groups. The statistical analyses were performed using SPSS 22.0 software. P-values ​​less than 0.05 were considered statistically significant in all cases.

## Results

In total, 666 cases were eligible for this study. The number of patients in Group A and B were 320 and 346 cases, respectively. Table 1 shows the demographic characteristic of patients in the two groups. All patients were followed for at least 2 years. At the 6-month follow-up, the success rate in Group A was 95.9% compared with 93.6% in Group B (P=0.2). The most common cause of failure in both groups was re-perforation (66.7% and 56.5%, respectively). At the 2-year follow-up, the success rate continued to decline and reached 91.6% and 93.4% in Group A and B, respectively (P=0.3). In general, the causes of failure in Group A were re-perforation (5.6%),cholesteatoma (2.5%), and severe retraction (0.3%). The most common causes of failure in Group B were re-perforation (3.8%), cholesteatoma (2.0%), and severe retraction (0.9%). The chance of anatomical success using the fascia temporalis graft with cartilage reinforcement was greater than with fascia temporalis only (odds ratio [OR]=1.3, 95% confidence interval [CI]: 0.7 to 2.4). In this study, age and sex had no significant effect on the success rate of surgery (P>0.05).

In this study, there was a significant reduction in ABG in both groups (P<0.001). In Group A, the ABG decreased from 28.1 ± 10.1 dB to 11.8 ± 8.9 dB. In 87.5% of the patients, the gap was less than 20 dB after surgery. In Group B, the ABG was 21.2 ± 8.6 dB pre-operatively and reached 17.3 ± 8. 1 dB after surgery. Post-operatively 55.5% of the patients showed ABG less than 20 dB. The difference between the rates of ABG improvement in the two groups was significant (P<0.001). In both groups, age, sex, and etiology of COM showed no effect on the auditory outcome (P>0.05).

**Table 1:** Demographic characteristics of patients with chronic otitis media undergoing type 1 tympanoplasty surgery using fascia temporalis only or fascia temporalis with cartilage reinforcement.

	Group A (Fascia temporalis only, n = 320)	Group B (Fascia temporalis & cartilage reinforcement, n = 346)
**Gender (%)**		
** Male** ** Female**	48.8 51.2	44.8 55.2
**Age (year; mean±SD)**	32.9±13.2	29.5±10.7
**Length of follow-up (month; mean±SD)**	24.7±6.3	26.3±5.5
**Air bone gap (dB; mean±SD)**	28.1±10.1	21.2±8.6

## Discussion

  Cartilage can be used as a graft to repair the tympanic membrane, especially when there is advanced ear pathology. Since cartilage has a higher rigidity than fascia, it is more resistant to absorption and retraction. These benefits have made cartilage popular as a graft. The cartilage graft can be used as perichondrium, perichondrium-cartilage, or cartilage. Also, various cartilage grafts can be used, such as island, palisade or cartilage reinforcement. In cartilage reinforcement, fascia temporalis is strengthened by applying cartilage graft in the superoposterior of graft which prevents occurrence of retraction pocket. In addition, cartilage reinforcement in the anterior part of the graft prevents medialization of fascia temporalis (8).

In this study, the success rate in the cartilage group was higher than in the fascia-only group (93.4% vs. 91.6%), and age and sex had no significant effect on the success rate of the surgery. This finding is in line with Kalcioglu study which showed no statistically significant difference between cartilage and fascia in the short-term and long-term follow-up (7). Although the remaining disease could be hidden in the epitympanum in the group undergoing fascia and cartilage reinforcement, we showed that failure due to cholesteatoma was similar in both groups. This finding is contrary to previous studies which showed that the usage of cartilage for total perforation has disadvantages and may hide cholesteatoma (11,12). Overall, our study shows hearing improvement in both groups. However, postoperative ABG less than 20 dB was seen in 87.5% of patients in Group A and 55.5% in Group B. This finding is consistent with the study of Gerber et al. which showed that reconstruction of the tympanic membrane with cartilage can impair auditory function (12). Some researchers such as Zahnert et al. and Murbe et al. believe that thinning of the cartilage up to 0.5 mm could confer acoustic benefits (13,14). However, we agree with Atef et al. that thinning of the cartilage is a technical problem (15), and does not lead to significant hearing impairment. We recommended a separate study comparing hearing between full thickness cartilage and partial thickness cartilage.

## Conclusion

In conclusion, the results of our study show that hearing improvement is lower with a fascia temporalis graft with cartilage reinforcement than with temporal fascia alone. However, the odds of achieving anatomical success are higher with cartilage reinforcement (OR=1.3).
